# SARS-CoV-2 mechanisms of cell tropism in various organs considering host factors

**DOI:** 10.1016/j.heliyon.2024.e26577

**Published:** 2024-02-20

**Authors:** Emad Behboudi, Seyed Nooreddin Faraji, Gholamreza Daryabor, Seyed Mohammad Ali Hashemi, Maryam Asadi, Fahime Edalat, Mohammad Javad Raee, Gholamreza Hatam

**Affiliations:** a Department of Basic Medical Sciences, Khoy University of Medical Sciences, Khoy, Iran; b Department of Pathology, School of Medicine, Shiraz University of Medical Sciences, Shiraz, Iran; c Autoimmune Diseases Research Center, Shiraz University of Medical Sciences, Shiraz, Iran; d Department of Bacteriology & Virology, Shiraz University of Medical Sciences, Shiraz, Iran; e Department of Microbiology, Golestan University of Medical Sciences, Gorgan, Iran; f Department of Molecular Medicine, School of Advanced Medical Sciences and Technologies, Shiraz University of Medical Sciences, Shiraz, Iran; g Center for Nanotechnology in Drug Delivery, Shiraz University of Medical Sciences, Shiraz, Iran; h Basic Sciences in Infectious Diseases Research Center, Shiraz University of Medical Sciences, Shiraz, Iran

**Keywords:** SARS-CoV-2, COVID-19, Entry route, Spike, ACE2

## Abstract

A critical step in the drug design for SARS-CoV-2 is to discover its molecular targets. This study comprehensively reviewed the molecular mechanisms of SARS-CoV-2, exploring host cell tropism and interaction targets crucial for cell entry. The findings revealed that beyond ACE2 as the primary entry receptor, alternative receptors, co-receptors, and several proteases such as TMPRSS2, Furin, Cathepsin L, and ADAM play critical roles in virus entry and subsequent pathogenesis. Additionally, SARS-CoV-2 displays tropism in various human organs due to its diverse receptors. This review delves into the intricate details of receptors, host proteases, and the involvement of each organ. Polymorphisms in the ACE2 receptor and mutations in the spike or its RBD region contribute to the emergence of variants like Alpha, Beta, Gamma, Delta, and Omicron, impacting the pathogenicity of SARS-CoV-2. The challenge posed by mutations raises questions about the effectiveness of existing vaccines and drugs, necessitating consideration for updates in their formulations. In the urgency of these critical situations, repurposed drugs such as Camostat Mesylate and Nafamostat Mesylate emerge as viable pharmaceutical options. Numerous drugs are involved in inhibiting receptors and host factors crucial for SARS-CoV-2 entry, with most discussed in this review. In conclusion, this study may provide valuable insights to inform decisions in therapeutic approaches.

## 1 Introduction

The emergence of pandemic infectious diseases poses a severe threat to global public health and the economy [1]. The recent COVID-19 pandemic recorded over 696 million cases and 6.9 million deaths (World Health Organization, COVID-19 situation reports). Preceding this, SARS-CoV (2002) and MERS-CoV (2012) were notable epidemics [2]. Current evidence indicates that SARS-CoV-2, causing the COVID-19 pandemic, exhibits higher severity and prevalence compared to MERS-CoV. The transmissibility of SARS-CoV-2 is greater than that of MERS-CoV in the Middle East but lower in the Republic of Korea. This variability may be attributed to mutations in host or virus proteins, diverse entry routes, and variations in host cell receptors [3,4]. SARS-CoV-2 primarily interacts with the angiotensin-converting enzyme 2 (ACE2) receptor, while MERS-CoV binds to the Dipeptidyl peptidase-4 (DPP4) receptor [[5], [6], [7]]. Docking studies reveals a significant interface between the SARS-CoV-2 spike glycoprotein and the DPP4 complex, indicating close similarity with other coronaviruses [8].

SARS-CoV and MERS-CoV, both members of the Coronaviridae family, share a positive-sense, single-stranded RNA genome. The genome of SARS-CoV-2 encodes 16 non-structural proteins, 4 structural proteins (Spike, Membrane, Envelope, and Nucleocapsid), and 9 accessory proteins [2,9]. Within the virus's structural proteins, Spike holds particular significance as it facilitates the interaction between the virus and host tissue receptors, leading to host invasion. The proteolytic activation of the spike, cleaving it into S1 and S2, is crucial and primarily governed by host cell proteases. Consequently, the pathogenesis of the coronavirus is fundamentally controlled by the interactions between host cell receptors and the spike protein [10,11]. The main role of the S1 subunit is to engage with the host cell's surface receptors through a specific receptor-binding domain (RBD), while the S2 subunit mediates the fusion of both virus-cell and cell-cell membranes [12].

Recent findings indicate the involvement of various molecules in the penetration of the SARS-CoV-2 virus into host cells. These include ACE2 (the most widely recognized), along with ASGR1, KREMEN1, AXL, CD147, and host cell proteases [13]. The clinical outcomes of COVID-19 exhibit variation among different populations and even within a single population. This suggests a genetic susceptibility in certain individuals to COVID-19 infection across diverse populations [14]. Polymorphisms in various receptors and co-receptors of SARS-CoV-2, influencing the virus's entry into diverse tissues among different individuals, may contribute to the observed clinical variability in the disease across subjects [15]. Unfortunately, the restricted understanding of the human molecular targets of SARS-CoV-2 hinders the development of host-dependent personalized therapies [16]. In this study, our objective is to bridge this knowledge gap by examining recent findings related to a crucial stage in viral infection, specifically the entry mechanisms of SARS-CoV-2. Our aim is to identify potential entry interactions, assisting researchers in designing effective prophylactic or therapeutic strategies. Additionally, we will provide a detailed discussion on the majority of molecules implicated in virus entry, along with an exploration of the organs involved in COVID-19 disease.

## 2 Methodology

Two investigators conducted separate searches for potentially eligible studies across databases such as PubMed, Scopus, Embase, and Web of Science (ISI) from inception to February 28, 2023. To pinpoint studies examining the interaction between the SARS-CoV-2 spike and human proteins, a combination of Mesh terms and keywords (SARS-CoV-2 OR Severe Acute Respiratory Syndrome Coronavirus 2) AND (Entry OR Route OR Mechanisms OR Receptor OR Interaction OR Attachment OR Binding) was applied. Additionally, a manual review of the references in the retrieved articles and relevant previous reviews as well as combination search with different human body organs was carried out to minimize the risk of overlooking studies. The management of records was facilitated using EndNote software, version X7. Following the removal of duplicates, two independent authors screened the search results based on title/abstracts and excluding irrelevant records. Studies focusing on polymorphisms or mutations in both the SARS-CoV-2 spike and human targets of the spike, including ACE2, TMPRSS2, Furin, and CD147, were selected.

## 3 SARS-CoV-2 spike glycoprotein and mutations affecting its binding

The spike glycoprotein plays a crucial role in the pathogenicity of SARS-CoV-2, encompassing virus attachment, fusion, and entry into the host cell [17,18]. The spike proteins undergo direct exposure to the host immune system and neutralizing antibodies [19]. The spike protein is initially synthesized as a precursor consisting of 1273 amino acids within the confines of the rough endoplasmic reticulum (RER). Subsequent to this synthesis, a series of modifications transpire in the Golgi complex, a process inclusive of cleavage into the S1 and S2 subunits facilitated by furin or furin-like proteases [20,21]. This cleavage event is orchestrated by the insertion of 12 nucleotides encoding four amino acid residues, specifically PRRA, demarcating the boundary between S1 and S2 [22,23]. The functional roles of these subunits are pivotal, with the S1 subunit binding the virus to the host receptor's surface, while the S2 subunit mediates the fusion between the virus and host cell membranes [24]. SARS-CoV-2 evolutionary modifies itself to evade the host immune system, introducing amino acid substitution in the Spike structure. Notably, the Asp614Gly substitution is implicated in influencing virus transmission speed [25]. Spike proteins have interaction with the positions 30–41 of the ACE2 protein [26]. The tight connection at the ACE2-SARS-CoV-2 interface is facilitated by three hydrogen bonds involving Gln493, Tyr449, and Gln498 of Spike, and Glu35, Lys353, and Asp38 of ACE2, along with a salt bridge formed between Asp30 of ACE2 and Lys417 of the spike [27]. The glycans are also essential for ACE2-virus surface interaction. They influence the conformation of the ACE2 receptor to allow the interaction of spike with ACE2 [28]. The glycans can modulate Spike-ACE2 interactions by masking polypeptide epitopes [29]. The negatively charged human cells bind to the positively charged O-linked glycan domain of the Spike [30]. The conformational dynamics of the RBD are modulated by N-glycans at sites N165 and N234, which play a crucial role in ACE2 recognition. A deletion in these regions can decrease the binding affinity of ACE2 [31]. ACE2-K26R mutation, observed in the Ashkenazi Jewish population, result in reduced electrostatic attraction of SARS-CoV-2/ACE2, while mutations D206G, I468V, G211R, R219C, and K341R increase it [32]. Furthermore, three missense changes N720D, Y26R, and G211R, common in the Italian population, are predicted to interfere with ACE2 protein stabilization [33]. Since the onset of the SARS-CoV-2 outbreak, the virus has undergone numerous mutations, resulting in various variants classified as Alpha (B.1.1.7 and Q lineages), Beta (B.1.351 and descendent lineages), Gamma (P.1 and descendent lineages), Epsilon (B.1.427 and B.1.429), Eta (B.1.525), Iota (B.1.526), Kappa (B.1.617.1), Mu (B.1.621, B.1.621.1), Zeta (P.2), Delta (B.1.617.2 and AY lineages) [34] and Omicron (strain B.1.1.529). First identified in South Africa on November 9, 2021, the Omicron variant has emerged as the sole current variant of concern. Notably, the Omicron variant demonstrates a transmission capacity 3–4 times higher than that of the delta variant, contributing to its rapid global spread and prevalence [35].

**Fig. 1:**
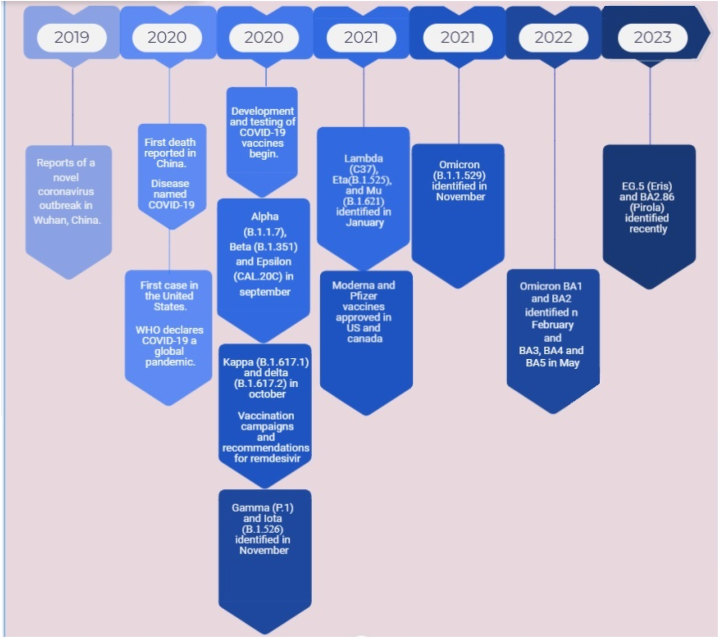
History of emergence of SARS-CoV-2.

Currently, there are several sub-variants of the Omicron. In comparison with the original Omicron BA.1, BA.2 displayed the higher resistance to available vaccines and this occurred in the next emerged variants. In 2023, a new Omicron variant called EG.5 (known as “Eris”) is the dominant variant in the U.S., and scientists are monitoring another new variant called BA.2.86 (also known as “Pirola”) (Fig. 1). Evidence has shown that this type of variant in some subjects causes more serious complications for young people, necessitating adjustments to health protocols to a certain extent [35,36].

These variants are known with the key mutations found in their spike which is listed below.

1. Alpha Variant (B.1.1.7):
  • Key mutation: N501Y, associated with increased binding affinity to the ACE2 receptor, possibly leading to increased transmissibility.
  • Other mutations: Del69-70, P681H.
2. Beta Variant (B.1.351):
  • Key mutation: E484K, associated with potential immune escape from neutralizing antibodies.
  • Other mutations: N501Y, K417 N.
3. Gamma Variant (P.1):
  • Key mutations: E484K, N501Y, and K417T, similar to the Beta variant.
  • Additional mutations: L452R.
4. Delta Variant (B.1.617.2):
  • Key mutations: L452R and T478K, associated with increased transmissibility and potential immune evasion.
5. Epsilon Variant (B.1.427 and B.1.429):
  • Key mutation: L452R.
6. Iota Variant (B.1.526):
  • Key mutations: E484K, S477 N, and D253G.
7. Kappa Variant (B.1.617.1):
  • Key mutations: E484Q and L452R.
8. Mu Variant (B.1.621):
  • Key mutations: E484K and N501Y.
9. Zeta Variant (P.2):
  • Key mutations: E484K and D614G.
10. Omicron Variant (B.1.1.529):
  • Key mutations: Omicron has a large number of mutations, including numerous changes in the spike protein (S gene), such as N501Y, E484A, K417 N, and many others.

In contrast to SARS-CoV, SARS-CoV-2 undergoes pre-activation by pro-protein convertases, particularly furin, facilitating cell entry. This characteristic reduces its reliance on target cell proteases for entry. Notably, the continuous variation in the SARS-CoV-2′ RBD results in a higher binding affinity to the host ACE2 compared to SARS-CoV, as the ACE2 site was less exposed to SARS-CoV’ RBD. These features contribute to efficient cell entry, evasion of immune surveillance, and viral widespread [37].

## 4 Human target proteins involved in spike interactions and drugs

### 4.1 ACE2; the major receptor

The aminopeptidase ACE2 acts as a functional receptor for the Spike of SARS-CoV-2 [38]. This protein plays a crucial role in regulating the renin-angiotensin-aldosterone system. The significance of this system in various physiological processes has sparked debate about targeting ACE2 for the treatment of COVID-19 [39]. The ACE2 regulation in human cells is associated with overexpression of some genes, including KDM5B, RAB1A, TLR3, HAT1, ADAM10, HDAC2, SIRT1, and FURIN, or downregulation of the TRIB3 gene [40]. The ACE2 gene is demonstrated to be expressed in approximately 72 human tissues, with its primary expression occurring at the surface of lung epithelial cells. Nevertheless, its protein has been identified in various other tissues, including the heart, kidney, testis, conjunctiva of the eye, cornea, interlobular pancreatic ducts, placental villi, cytotrophoblasts, syncytiotrophoblasts, extravillous trophoblasts, and at the base of ciliated fallopian tube epithelium [41,42].

As age progresses, the expression of ACE2 within the alveolar epithelial cells of the lungs, cardiomyocytes, and vascular endothelial cells increases, contributing to an age-related susceptibility to the virus [43]. It has been demonstrated that ACE2 gene polymorphisms might increase the susceptibility of individuals to SARS-CoV-2 infection, and its complexities including hypertension, multi-organ failure, and cardiovascular and pulmonary conditions [44,45]. Amino acid substitution in the human ACE2 protein, such as K26R, I21V, E23K, S19P, T27A, T92I, Q102P, H378R, and N64K, have been linked to an increased susceptibility to SARS-CoV-2. Conversely, other ACE2 residue substitutions, including K31R, E35K, N33I, H34R, M62V, E37K, D38V, Y50F, D355 N, N51S, K68E, Y83H, G326E, G352V, F72V, Q388L, and D509Y, are considered protective mutations due to their association with reduced binding affinity to the Spike protein [44,46].

Notably, the N720D variant in the C-terminal Colectin-like domain (CLD) of ACE2 has the potential to impact its stability and flexibility. This alteration further creates a more favorable site for TMPRSS2 binding, Spike protein cleavage, and subsequent viral entry [47]. The RBD segment which is located within the residues 318–510 of the Spike protein, undergoes frequent mutations and plays a pivotal role in facilitating virus attachment to ACE2 [48]. During the initial fusion process between the Spike and ACE2 receptor, only 27 residues (13 in Spike, 14 in ACE2) are actively involved [49]. However, certain mutations in the RBD of SARS-CoV-2 have been identified to enhance its affinity for ACE2, consequently increasing its infectiousness [50]. In-silico models have proposed that targeting the hot spot 353 residue location to inhibit the recognition of Spike by ACE2 could be an effective strategy to prevent SARS-CoV-2 infection [51]. Interestingly, in a phase II clinical trial, the administration of recombinant human ACE2 demonstrated a reduction in systemic inflammation among COVID-19 patients [52]. Molecular docking studies have revealed that remdesivir and ribavirin exhibit higher molecular binding energies to ACE2 [53]. Additionally, Sphingosine has been shown to prevent the interaction between ACE2 and the RBD segment [54]. Certain allelic variants of the ACE2 gene, such as rs73635825 and rs143936283, provide a structural basis for potential resistance against SARS-CoV-2 infection mediated by ACE2 [38]. Garcinia biflavonoid-2 (GB-2), containing flavin, has demonstrated the ability to inhibit the expression of ACE2 and TMPRSS2 genes both in vitro and in vivo without cytotoxicity, making it a potential candidate drug for preventing SARS-CoV-2 infection [55]. In a study conducted in 2023, Wu et al. found that GB-2 reduced the entry efficiency of the Omicron variant (BA.1) of SARS-CoV-2 with the N501Y, K417 N, E484A, G339D, Q493R, G496S, Q498R mutations and inhibited the binding between ACE2 and the RBD [56]. Soluble ACE2 isoforms, generated by removing specific exons in the 3′UTR of ACE2 through alternative splicing, suggest that Splice-switching antisense oligonucleotides (SSOs) could serve as a therapeutic strategy [57].

### 4.2 Alternative SARS-CoV-2 receptors and co-receptor

Due to the diverse organ tropisms observed in SARS-CoV-2 infections involving various organs, the virus utilizes multiple receptors and co-receptors [18]. Apart from ACE2, researchers has explored alternative molecules that may function as receptors for SARS-CoV-2, including ASGR1, KREMEN1, and AXL [13,58]. A study by Gu et al. demonstrated elevated expression of ACE2, KREMEN1, and ASGR1 receptors in airway epithelial secretory cells and macrophages [59]. Furthermore, investigations indicated that the combined function of these three molecules ASGR1, KREMEN1, and AXL (ASK) exceeds that of individual molecules, potentially contributing to the complexity of COVID-19 infections and their associated complications [59]. The tyrosine-protein kinase receptor UFO, known as AXL, plays a crucial role in SARS-CoV-2 infection through its interaction with the N-terminal domain of the Spike protein in human primary lung epithelial cells [60]. Given the impact of the AXL receptor on COVID-19 infection, the AXL inhibitor bemcentinib has been recently explored as a potential treatment and is currently in phase II trials [61]. In vitro experiments have demonstrated the efficacy of reducing viral infection in pulmonary epithelial cells by either knocking down Axl or adding it in soluble recombinant form to the cell culture. Interestingly, the natural ligands of Axl, Gas6 and protein S, do not bind to SARS-CoV-2 [62].

Aminopeptidase N (APN) is a member of zinc peptidase family which acts as the receptor for several coronaviruses, especially 229E, an enteropathogenic coronaviruse [63]. Blocking APN have been revealed as a prophylaxis strategy in high-risk individuals infected by SARS-CoV [64]. However, the specific role of APN in SARS-CoV-2 pathogenesis remains undetermined. Dipeptidyl peptidase 4 (DPP4) and glutamyl aminopeptidase (ENPEP) were identified as the candidate co-receptors for human SARS-CoV-2 [65,66]. Additionally, integrin has been proposed as an alternative receptor for the virus, potentially influencing its transmission and pathology [67]. Given the presence of the integrin-binding motif on the surface of the Spike, testing Integrin binding experimentally has been suggested as a potential therapeutic target [56]. Recent findings also indicate that CD147 (basigin) may serve as an additional entry point for SARS-CoV-2 infection through interaction with the viral spike [68].

### 4.3 Human host cell proteases

#### 4.3.1 Transmembrane protease serine or TMPRSS family (TMPRSS2, TMPRSS4)

**Fig. 2:**
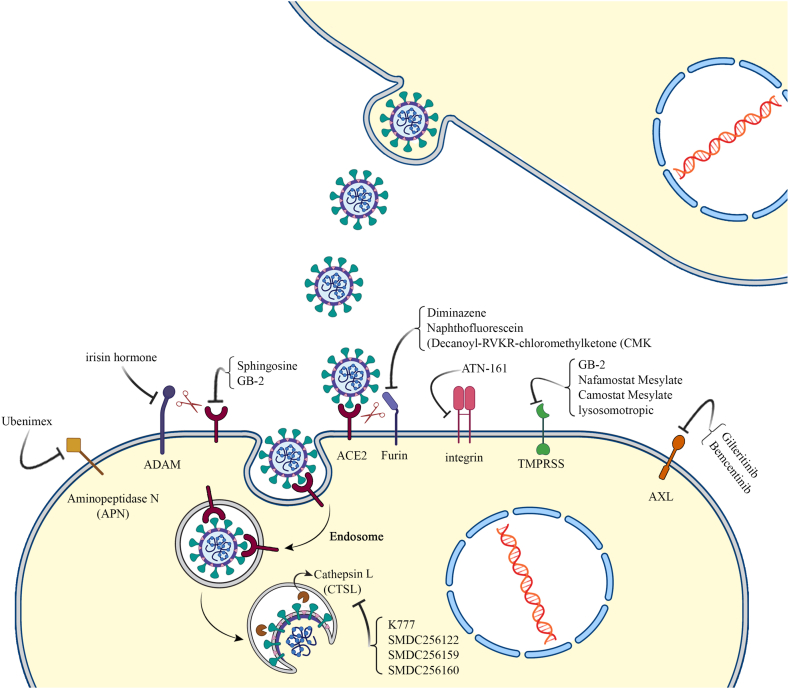
SARS-CoV-2 receptors and co-receptors on the surface and in the host cells and their potent inhibitors.

In SARS-CoV-2 entry, early pathway is used in the presence of TMPRSS2. ACE2 can be shed by ADAM17 and TMPRSS2 proteases [69]. TMPRSS2 cleaves and activates the Spike of coronaviruses es well as SAR-CoV-2 at S29 location, thereby leading to substantial structural rearrangement for ACE2 binding and membrane fusion viral entry [70]. TMPRSS2 is primarily expressed in bronchial transient secretory cells [71] and its inhibition has been shown to prevent certain coronaviruses from entering host cells [72]. Interestingly, smoking has been indicated to increase expression levels of TMPRSS4 in the bronchial epithelial cells, rendering smokers more susceptible to the SARS-CoV-2 infection [73]. Both TMPRSS2 and TMPRSS4 can further enhance SARS-CoV-2 infection in human small intestinal enterocytes [74]. Several genetic variants from TMPRSS2 genes, including rs112657409, rs11910678, rs61735792, rs61735794, rs75603675, rs77675406, rs464397, rs469390, rs2070788, rs383510, and rs713400, have been identified as potential influencers of TMPRSS2 expression levels, subsequently altering individuals' susceptibility to SARS-CoV-2 infection [[75], [76], [77]]. Approved TMPRSS2 inhibitors, including nafamostat mesylate and camostat mesylate are under clinical trials and caused reduced death rate [78] (Table 1 and Fig. 2).

**Table 1:** Shows human target proteins involved in spike of SARS-CoV-2 interactions and their inhibitors.

Function	Protein	Candidate drugs for blocking	Reference
**Receptors**	ACE2	Sphingosine, GB-2	[56,79,80]
	ASGR1	Not currently available	
	KREMEN1	Not currently available	
	AXL	Gilteritinib, Bemcentinib (in phase II trials)	[62,81,82]
	Aminopeptidase N (APN)	Ubenimex (its derivatives)	[83]
	integrin	ATN-161	[84]
**Co-receptors**	Dipeptidyl peptidase 4 (DPP4)	Not currently available	
	glutamyl aminopeptidase (ENPEP)	Not currently available	
	CD147 (basigin)	Not currently available	
**Human host cell proteases**	TMPRSS family	GB-2, Nafamostat Mesylate, Camostat Mesylate, lysosomotropic	[78,80,85]
	Furin	Diminazene, Naphthofluorescein, Decanoyl-RVKR-chloromethylketone (CMK)	[86,87]
	A Disintegrin and Metalloproteinase (ADAM)	irisin hormone	[40,88]
	Cathepsin L (CTSL)	K777, SMDC256122, SMDC256159, SMDC256160, chlorpromazine, fluoxetine and Active-6	[89,90,91,92]

#### 4.3.2 Furin

The serine proteases, notably Furin, and related pro-protein convertases (PCs) such as PC2, PC1/3, PC4, PACE4, PC5/6, and PC7 cleave Spike at the S1/S2 site, making them essential for viral entry [93]. In the case of MERS-CoV, Furin and other pro-protein convertases process the S1/S2 site, with the S2 site cleaved upon viral entry [94]. It has been indicated that the Spike of SARS-CoV-2 contains a furin cleavage site (PRRA) that is not detectable in other SARS-like CoVs [95]. The PRRA site promotes viral entry and cell to cell fusion (syncytium formation) but is not critical for infection [96,97]. Some scientists believe that furin-mediated cleavage at the modified R667 position of the Spike enhances membrane fusion activity without affecting virion entry [98]. The variants of SARS-CoV-2 lacking furin cleavage site have shown reduced replication in human respiratory cell lines [99]. Interestingly, the SARS-CoV-2 Spike protein mutant D614G, which is a virulent strain of SARS-CoV-2, has a higher affinity PRRA site for furin [100,101]. The use of furin inhibitors such as diminazene, naphthofluorescein, and decanoyl-RVKR-chloromethylketone (CMK) has been proposed to block the cleavage of the SARS-CoV-2 Spike protei and subsequently reducing virus replication and cytopathic effects [102,86].

#### 4.3.3 A disintegrin and metalloproteinase (ADAM)

ADAM, a family of transmembrane proteins, is widely expressed in various tissues including vascular smooth muscle cells, lung macrophages, and bronchial epithelial cells. ADAM proteins, such as ADAM-17, are responsible for cleaving the ectodomains of transmembrane proteins like ACE2. Stimulating ADAM-17 can increase ACE2 shedding, making it an appealing target for pharmacologic intervention [103,104]. There is a suggested association between ADAMTS13, a protein containing a thrombospondin type1 motif, member 13, and the promotion of SARS-CoV-2 pathogenesis. Reducing ADAMTS13 levels has been proposed as a strategy to lower COVID-19 mortality [105,106]. Additionally, the hormone Irisin, secreted during exercise, has been found to modulate the expression of ADAM genes [40]. In a 2023 trial, Alves et al. demonstrated that Irisin may also mitigate excessive inflammation associated with COVID-19, potentially reducing the severity of the disease's outcomes [88].

#### 4.3.4 Cathepsin L (CTSL)

Cathepsin L (CTSL) is activated in the low-pH environment of the endosome and triggers late pathways [107]. As a lysosomal cysteine protease, CTSL plays a crucial role in cleaving the S1 subunit of the Spike protein, necessary for viral entry, virus and endosomal membrane fusion, and viral RNA release for the next round of replication [89]. CTSL inhibitors have been demonstrated to block the entry of SARS-CoV, indicating the virus utilizes CTSL to infect ACE2-expressing cells [108,109]. Following SARS-CoV-2 infection, circulating CTSL levels increase, and higher levels are associated with disease severity [110]. CTSL inhibitors such as K777 and its derivatives, including SMDC256122, SMDC256159, and SMDC256160 have successfully inhibited virus entry into the host cells. However, the simultaneous use of serine protease and CTSL inhibitors is suggested as a potentially safer and much more effective therapy compared to other available therapies for blocking viral entry [111]. Lysosomotropic cathepsin L inhibitors, including chlorpromazine and fluoxetine, are under clinical trials [90]. In an in vitro experiment, fluoxetine demonstrated successful inhibition of SARS-CoV-2 gene expression. Additionally, fluvoxamine (Active-6) may exert an immunomodulatory effect by strongly binding to the sigma 1 receptor (S1R), known for significant roles in immune system modulation and suppression of inflammatory cytokines. Both fluoxetine and fluvoxamine can influence intravesicular pH, hindering viral compartment fusion and limiting internal virus spread. Consequently, the study have reported that fluoxetine can reduce mortality associated with COVID-19 infection [91].

#### 4.3.5 Latest drugs in clinical trials

In addition to the ongoing exploration of novel treatment modalities involving new pharmaceuticals, immunotherapeutic agents, and host-directed therapeutic approaches, the global scientific community is actively investigating the potential effectiveness of pre-existing drugs against SARS-CoV-2. Our search on ClinicalTrials.gov has identified numerous clinical trials dedicated to evaluating drugs for COVID-19 treatment. These drug candidates can be broadly categorized into two main groups: those designed to directly impede the viral replication cycle, and those employing immunotherapy strategies to bolster the innate antiviral immune responses or mitigating damage triggered by dysregulated inflammatory reactions. Some of these newest drugs with promising outcomes are DFV890 (Phase II), TD-0903 (Phase II), RBT-9 (Phase II), M5049 (Phase II), SNG001(Phase II), COVID19-0001-USR (Phase II), LAU-7b (Phase II), and BLD-2660 (Phase II), and Paxlovid (phase III/IV) [112,113].

## 5 SARS-CoV-2 tropism and entry into different human tissues

About 15–20% of the SARS-CoV-2 patients have lung involvement, 10% have asymptomatic infection, and 5–10% develop multiple organ failure [114,115]. Investigations into SARS‐CoV‐2 entry mechanisms have predominantly concentrated on ACE2 and priming proteases, both of which exhibit notably low expression levels, especially in respiratory epithelial cells. This prompts the consideration that additional cofactors might be necessary to aid SARS‐CoV‐2 entry into cells with limited ACE2 expression. A study on normal human tissues reported ACE2 as the central receptor and neuropilin-1, GRP 78, CD147 and other SARS-CoV-2 receptors expressed at a different levels in various organs [116,117]. SARS-CoV-2 nucleocapsid has been identified in a range of cell types, showing a strong colocalization with various cellular components, including mitochondrial proteins, lipid droplets (LDs), and several host proteins known for their implications in inflammation, tissue repair, and the SARS-CoV-2 life cycle. These host proteins contain vimentin, fibronectin, NLRP3, LC3B, DDX3X, and PPARγ, which their potential role in the interplay between viral infection, inflammation, and pathogenesis has been evaluated [115]. SARS-CoV-2 targets different cells in various tissue types, including lung epithelial and endothelial cells, as well as lipogenic fibroblast-like cells (FLCs) exhibiting characteristics similar to lipofibroblasts. These cells feature activated PPARγ signaling and the presence of lipid droplets (LDs) [118]. Furthermore, potential targets of SARS-CoV-2 include lung fibroblast-like cells (FLCs) expressing fibronectin and vimentin, macrophages exhibiting NLRP3- and IL1β-induced responses, regulatory cells with immune-checkpoint proteins involved in lung repair and contributing to inflammatory responses, CD34^+^ liver endothelial cells and hepatocytes expressing vimentin, renal interstitial cells, and the juxtaglomerular apparatus. These diverse cell types highlight the virus's ability to interact with various tissues and cell populations within the body [119]. These observations suggest that SARS-CoV-2 directly impacts crucial functions in the lungs, kidneys, and liver, implicating these organs in COVID-19 pathogenesis. The intricate interactions between the virus and various cellular components highlight the complexity of its actions within the human body, potentially influencing disease severity. Patients with COVID-19 have experienced a range of complications, including myocarditis, encephalopathy, Prion-like disease, Guillain-Barré syndrome, thrombotic thrombocytopenia, and mucormycosis, both during and after the acute phase. Certain symptoms during the acute phase, such as vomiting, throat pain, diarrhea, gastrointestinal symptoms, dyspnea, or headache, may be associated with the development of long-term symptoms known as long COVID [23,24]. In-depth analysis reveals that these symptoms, as well as cough, muscle pain, and weakness, are common among both hospitalized and non-hospitalized long COVID patients. Moreover, individuals who experienced mental health issues diagnosed during the acute phase of COVID-19 faced an increased risk of developing long COVID. Recent analysis of electronic health records from primary care practices in the UKsupports this finding, indicating that conditions like dementia and impaired mental health in COVID-19 survivors pose significant risk factors for complications and mortality compared to patients hospitalized for influenza. These associations underscore the potential psychobiological mechanisms linking COVID-19 to neurological damage and the development of persistent long COVID symptoms [25].

### 5.1 Lung

The airway epithelial cells and lung alveoli express low levels of ACE2 but elevated levels of TMPRSS2, GRP78, and CD147 that facilitate the entrance of the SARS-CoV-2 [120]. The main target cells for SARS-CoV-2 are Human alveolar epithelial cells [121]. Interacting with ACE2, SARS-CoV-2 can disrupt the renin-angiotensin-aldosterone system and activates certain cell signaling pathways that are related to lung fibrosis [122].

### 5.2 Cardiovascular

Cardiac damage in SARS-CoV-2 infection has been attributed to various mechanisms, including Spike-ACE2 interaction, cytokine storm, and hypoxemia [123]. While the ACE2 gene is more highly expressed in the human heart than the lung, TMPRSS2 is rarely expressed. The minimal coexpression of ACE2 and TMPRSS2 genes in cardiac cells suggests an alternative viral entry mechanism in SARS-CoV-2 infection within the heart. It is plausible that the virus utilizes other entry factors, such as cathepsin L, which are notably abundant in cardiomyocytes, rather than relying on the TMPRSS2-dependent pathway. This highlights the intricate ways in which SARS-CoV-2 may affect cardiac tissues [124]. Analysis of heart tissue from COVID-19 patients reveals changes in cell composition, including a significant decrease in cardiomyocytes and pericytes, along with an increase in vascular endothelial cells. Notably, viral RNA is not detected in these cell types. Reports suggest the presence of infected nonmuscle cells in COVID-19 cardiac tissue, while another report indicates SARS-CoV-2 antigen in cardiomyocytes, suggesting potential direct myocardial damage [125]. Human induced pluripotent stem cell (hiPSC)-derived heart cells show susceptibility to SARS-CoV-2 infection, resulting in myofibrillar fragmentation and nuclear disruption, potentially linked to dysregulation of genes associated with the nucleoskeleton-cytoskeleton complex. This complex interplay also sheds light on mechanisms underlying COVID-19-related cardiac complications [126].

### 5.3 CNS

The neurotropic capability of SARS-CoV-2 mainly arise from its binding potential to the transmembrane receptor neuropilin-1 (NRP1), which is highly expressed in the olfactory epithelium, olfactory tubercles, and paraolfactory [127]. The virus is thought to potentially spread to the central nervous system (CNS) via the olfactory nerve [128]. There are several studies reported CNS complications such as encephalopathy, progressive dementia, Prion-like disease, Guillain-Barré syndrome after COVID-19 acute infection and vaccination [23,24]. Recent preliminary findings also indicate that the Spike might have a binding affinity to EGFR, c-MET, and VEGFR on glioma cells [129]. Moreover, whole RNA sequencing revealed extensive expression of SARS-CoV-2, coronavirus-associated factors, and receptors in the human dorsal root ganglion (DRG) at the lumbar and thoracic levels. Nociceptors expressing MRGPRD mRNA, which also express ACE2 mRNA, may provide a route for SARS-CoV-2 entry into neurons forming free nerve endings in the outermost layers of the skin and luminal organs. Immunohistochemical analysis demonstrated ACE2 expression in the limbus, conjunctiva, and cornea [130]. Although the oral cavity might be considered a potential entry point for the virus, the presence of protease inhibitors in saliva should also be taken into account, indicating a complex interplay of factors influencing viral entry [131]. Certain mutations in SARS-CoV-2 enhance the Spike protein's activity, modifying its structure for increased binding to host cells. The impact on SARS-CoV-2 neuroinvasion is unclear. TMPRSS2 and neuropilin-1 are present in the central and peripheral nervous systems. Potential CNS entry pathways include infected immune cells crossing the blood-brain barrier, entry into cerebrospinal fluid via the choroid plexus, *trans*-synaptic viral transmission from various nerves, and access to circumventricular organs lacking the BBB. ACE2 and neuropilin-1 in retinal cells and the visual system are also considered potential entry points for SARS-CoV-2 invasion [132].

### 5.4 Gastrointestinal tract

Active viral replication has been confirmed in both the small and large intestines, as evidenced by electron microscopy, and viral genomes have been detected in stool samples. Enterocytes, rich in ACE-2 receptors and furin (a serine protease), facilitate virus entry, with TMPRSS2 and TMPRSS4 expression promoting this process [74,133,134]. ACE2-expressing cells are over tenfold more prevalent in the lower gastrointestinal tract (ileum, colon, and rectum) compared to the upper gastrointestinal tract (esophagus, stomach, and duodenum). Coexpression of ACE2 and TMPRSS2 is more common in the lower gastrointestinal tract. The SARS-CoV-2 nucleocapsid protein has been identified in the exocrine cells of the pancreas [135].

### 5.5 Other SARS-CoV-2 target organs or tissues

SARS-CoV-2 entrance into podocytes and tubular epithelial cells has been associated with renal abnormalities, including hematuria, proteinuria, and acute kidney injury [136]. Reports indicate that 36–46% of COVID-19 patients experience acute kidney damage (AKI), while severe cases may also involve liver damage. Both liver cells and bile duct cells express ACE2, with higher expression levels observed in bile duct cells [137,138]. ACE2 and TMPRSS2 are found in trophoblast, hypoblast, and syncytiotrophoblast tissues, raising the possibility of vertical SARS-CoV-2 transmission [139]. ACE2 is expressed in endothelial progenitor cells (EPCs) and hematopoietic stem cells (HSCs), potentially posing a risk of infection and damage to stem cells [140]. ACE2 and TMPRSS2 are present in a small population of cells in human umbilical cord blood (UCB) that can differentiate into functional EPCs and HSCs [140]. Band3 protein on the surface of red blood cells (RBCs) is considered a potential entry point for SARS-CoV-2. Binding of SARS-CoV-2 Spike to ACE2 on platelets induces platelet activation, possibly contributing to thrombus formation and inflammatory responses in COVID-19 disease [141,142].

## 6 SARS-CoV-2 and ACE2 expression in different ages, genders and ethnicities

Several factors, including age, gender, and ethnicity, may influence the outcome of COVID-19 disease. The location of the ACE2 gene on the X-chromosome suggests potential heterozygosity in females, differing from homozygous males [15,143]. Studies indicate that males have a higher susceptibility to SARS-CoV-2 in terms of mortality and the need for intensive care units. Genetic variations in essential genes like ACE2 and TMPRSS2, along with differences in immune system function, may contribute to the varying incidence and outcomes across ethnic groups [144]. Older patients with COVID-19 often face more severe disease. This may result from inadequate immune responses and higher underlying diseases. Increased expression of SARS-CoV-2 target proteins and heightened cell adhesion and stress responses contribute to this vulnerability. Additionally, reduced mitochondria and cellular replication, decreased proliferating natural killer and T cells, and increased endothelial cells and IGSF21+ dendritic cells further impact the severity in the elderly [[145], [146], [147]].

## 7 Pathogenesis of SARS-CoV-2 in individuals with pre-existing health conditions

Patients with underlying medical conditions, including cancer, diabetes, chronic kidney and obstructive pulmonary disease, and heart conditions, as well as those in an immunocompromised state, face an altered risk of severe COVID-19 infection. Higher mRNA and protein levels of ACE2 are observed in individuals with fundamental heart failure, suggesting an increased likelihood of severe COVID-19 in patients with primary cardiovascular disease [148]. Diabetic patients, due to the involvement of the PPAR signaling pathway in SARS-CoV-2 intrusion through the ACE2 receptor, exhibit greater vulnerability to COVID-19 infection [16,149]. Moreover, pancreatic islets of type 2 diabetes mellitus patients show significantly elevated ACE2 expression compared to healthy subjects [150]. Interestingly, cancer tissues, particularly in head and neck squamous cell carcinoma (HNSCC) and lung cancers, demonstrate lower ACE2 and TMPRSS2 expression, potentially rendering them more resistant to SARS-CoV-2 infection [151].

## 8 SARS-CoV-2 future prospective

A comprehensive search for human interaction targets in SARS-CoV-2 entry holds significant potential to identify host proteins influencing virus tropism in diverse tissues. Therapies targeting the host-virus interaction may offer durable and broad-spectrum treatment options [152]. Key players such as ACE2, TMPRSS2, CTSL, CTSB, Furin, ADAM, heparan sulfate, and CD147 play crucial roles in SARS-CoV-2 infectivity and entry. Understanding their expression patterns can shed light on viral tropism and its impact on the severity of the disease [[153], [154], [155], [156], [157]]. Furthermore, investigations into cell-type-specific associations with sex, age, and smoking provide valuable insights into the expression levels of ACE2, TMPRSS2, and CTSL [158]. ACE2, functioning as a regulator of the renin-angiotensin-aldosterone system, also serves as a functional receptor for SARS-CoV-2. The enriched interaction of proteins in the lung, particularly at higher expression levels, supports the idea that the virus targets proteins abundant in this tissue. The increased vulnerability of ACE2-expressing organs highlights its crucial role in the virus's entry, especially in older individuals with elevated ACE2 expression [159,160]. This understanding has led to the proposal of repurposing drugs, including recombinant soluble ACE2, TMPRSS2 inhibitors (camostat mesylate, nafamostat mesylate, antiandrogens, inhaled corticosteroids), and ADAM-17 enhancers (5-fluorouracil), along with indirect ACE2 modulators (angiotensin receptor blockers, calmodulin antagonists, and selective estrogen receptor modifiers) for targeting SARS-CoV-2 based on spike-host cell interactions [103].

Potential anti-viral therapies for SARS-CoV-2 include the application of anti-ACE2 antibodies or soluble ACE2 recombinant proteins. Inhibitors targeting ACE2, TMPRSS2, cathepsin, trypsin, furin, plasmin, factor X, and elastase show promise [54,[161], [162], [163]]. Other inhibitors, including hydroxychloroquine, hepcidin, Eltrombopag, IPB02, keto, and enol forms of curcumin, nelfinavir, cholesterol 25-hydroxylase, and various drugs like memantine, procyanidin, remdesivir, oseltamivir, and more, demonstrate potential in preventing viral entry or function [[164], [165], [166], [167], [168], [169], [170], [171], [172]]. Several compounds, including memantine, procyanidin, remdesivir, oseltamivir, zanamivir, Arbidol, Triazavirin, zanamivir, Mitoguazone, metformin, biguanide hydrochloride, gallic acid, caffeic acid, sulfaguanidine, heparin oligomers, riboflavin, fenoterol, cangrelor, hesperidin, vidarabine, ritonavir, and acetylcysteine, show potential as inhibitors against SARS-CoV-2, preventing virus fusion to host cells or viral entrance [[173], [174], [175], [176], [177], [178], [179], [180], [181], [182]]. Compounds like digitoxin, diammonium, ivermectin, rapamycin, rifaximin, amphotericin B, and glycyrrhizinate show desirable features and can be considered for COVID-19 therapies [183]. A recent study proposed amantadine as a therapeutic choice because it blocks the viroporine channel of COVID-19 and inhibits the release of the viral nucleus into the cytoplasm [184]. Besides lopinavir and ritonavirm, other protease inhibitors such as Nafamostat (anticoagulant), Phillyrin and chlorogenic acid effectively can prevent SARS-CoV-2 entry by inhibiting Spike protein-mediated fusion [185,186]. Vitamin D affects the Renin-Angiotensin system, preventing inflammation and lung injury. Interleukin 7 (IL-7) aggregation induces ACE2 expression, and vitamin C can block IL-7-induced ACE2 expression [43,187]. Drugs targeting lipid metabolisms, such as statins, triacsin, orlistat, and VPS34 inhibitor, may reduce disease severity [[188], [189], [190]]. Furthermore, Natural small molecules like cyclodextrin and sterols might inhibit the attachment to host cells and reduce viral infectivity [191].

## 9 Conclusion

In the present study, a comprehensive and systematic review was performed to identify most of the SARS-CoV-2 targets. The detailed knowledge of biological interactions is of utmost importance while designing new drugs or virtual screening of ligand databases against a particular target. Design of experimental or in-silico interaction analysis of the mentioned targets may shed more light on developing safe and effective anti-COVID-19 therapy.

## Data availability statement

Data included in the text, tables, figures, and referenced in the article.

## CRediT authorship contribution statement

**Emad Behboudi:** Writing – original draft, Validation, Conceptualization. **Seyed Nooreddin Faraji:** Validation, Supervision, Conceptualization. **Gholamreza Daryabor:** Writing – original draft, Investigation. **Seyed Mohammad Ali Hashemi:** Writing – review & editing, Validation. **Maryam Asadi:** Writing – original draft. **Fahime Edalat:** Writing – review & editing, Validation. **Mohammad Javad Raee:** Writing – review & editing. **Gholamreza Hatam:** Writing – review & editing, Validation, Conceptualization.

## Declaration of competing interest

The authors declare that they have no known competing financial interests or personal relationships that could have appeared to influence the work reported in this paper.
